# Meta-analysis of the prognostic value of CpG island methylator phenotype in rectal cancer

**DOI:** 10.1007/s00384-018-3108-5

**Published:** 2018-06-20

**Authors:** R. F. Kokelaar, H. Jones, J. Beynon, M. E. Evans, D. A. Harris

**Affiliations:** ABMU Singleton Hospital, Sketty Lane, Swansea, Wales SA2 8QA UK

**Keywords:** Methylation, CIMP, Rectal cancer, Biomarkers

## Abstract

**Purpose:**

The pathological and prognostic importance of CpG island methylator phenotype (CIMP) in rectal cancer, as a sub-population of colorectal cancer, is unknown. A meta-analysis was preformed to estimate the prognostic significance of CIMP in rectal cancer.

**Methods:**

A systematic search was performed of PubMed, Embase, MEDLINE, PubMed Central, and Cochrane electronic databases for articles pertaining to CIMP and rectal cancer. Articles were analysed and data extracted according to PRISMA standards.

**Results:**

Six studies including 1529 patients were included in the analysis. Following dichotomisation, the prevalence of CIMP-positive tumours was 10 to 57%, with a median of 12.5%. Meta-analysis demonstrated the pooled odds ratio for all-cause death for CIMP-positive tumours vs CIMP-negative tumours was 1.24 (95% CI 0.88–1.74). *Z* test for overall effect was 1.21 (*p* = 0.23). Heterogeneity between the studies was low (*X*^2^ 5.96, df 5, *p* = 0.31, *I*^2^ = 16%). A total of 15 different loci were used for assessing CIMP across the studies, with a median of 6.5 loci (range 5–8).

**Conclusions:**

No significant association between CIMP and poor outcomes in rectal cancer was demonstrated. There was a high degree of heterogeneity in CIMP assessment methodologies and in study populations. Rectal cancer datasets were frequently not extractable from larger colorectal cohorts, limiting analysis.

## Introduction

Colorectal cancer (CRC) is a significant health problem due to high prevalence and mortality, representing the third most common cause of cancer death in the USA [1]. Rectal cancer (RC) as a sub-population of approximately 30% of CRC poses additional significant mortality, morbidity, and management challenges, due to the anatomical confines of the bony pelvis and proximity of significant neurovascular structures and other organ systems [2]. The management of rectal cancers is currently undergoing a paradigm shift; previously inoperable locally advanced and locally recurrent rectal cancers are increasingly rendered operable due to improved operative techniques and the judicious use of neoadjuvant chemoradiation (nCRT) [3]. The potential for organ preservation is also increasingly accepted due to the introduction of minimally invasive surgery and ‘watch-and-wait’ strategies of non-operative management [4–7]. Despite advances, there is however an inability to determine which patients may benefit from one treatment modality or another based upon anything other than traditional radiological and histopathological staging [8, 9]. As the era of personalised medicine advances, there is an increasing need for molecular biomarkers that will aid decision making in the preoperative phase.

The molecular and genetic aberrations that underlie CRC carcinogenesis are complex and not fully understood, although there is a consensus that there are divergent processes responsible for tumour development at different sites throughout the colon and rectum [10]. DNA methylation is one epigenetic process implicated in CRC, as well as other cancers, and CpG island methylator phenotype (CIMP) has drawn interest as a potential mechanism underlying both carcinogenesis and as a potential biomarker [11, 12]. CIMP, however, has primarily been associated with carcinogenesis in the right colon that is characterised by hypermethylation and microsatellite instability (MSI) (the serrated pathway), rather than the traditional chromosomal instability pattern typical of other sites in the colon and rectum [13–15]. Despite the preponderance in the right colon, CIMP tumours are known to occur in the rectum, although the clinical significance of this molecular tumour type occurring at this site is poorly understood, although some authors have suggested they represent a poor-prognostic subgroup [16, 17]. Many of the studies that have examined the role of methylation in RC or indeed CRC have focused on a single gene locus or have relied upon small cohorts, making outcomes interpretation challenging [18, 19]. The aim of this paper is to review and meta-analyse the prognostic value of CIMP in adenocarcinoma in the rectum.

## Methods

### Search protocol

An online search was performed to retrieve original research articles where CIMP was assessed in rectal adenocarcinoma specimens, and where outcomes data were assessed (disease-free and overall survival (DFS and OS)). PubMed, Embase, MEDLINE, PubMed Central, and Cochrane databases were searched using the Boolean terms (CpG island methylator phenotype OR CIMP) AND (cancer OR carcinoma OR adenocarcinoma OR tumor OR tumour) AND (colorectal OR rectal) AND (prognosis OR outcome). A cutoff for inclusion was January 2018, results were compiled in a reference manager, and duplicates were removed. The grey literature was examined for additional contributions. Study design and search strategy was registered pre-emptively at PROSPERO (registration number CRD42018099569) [20].

### Study selection

All types of study were included in the analysis, although reviews, meta-analysis, and book chapters were excluded. Exclusion criteria were single-locus or gene methylation studies, studies where DFS and OS outcomes were not reported, and studies where the rectum was not defined as in identifiable cohort within colorectal cancers. Studies where surgery was not performed with curative intent were also excluded.

### Data extraction

Two reviewers independently applied the exclusion criteria to retrieved abstracts, and discrepancies were agreed by consensus. Data was extracted by one author from full-text manuscripts, and each dataset was verified by an independent reviewer. Baseline data for each study included author, date, institution, country, total number of patients, sex, TNM staging, method of determining CIMP, and methodology. Numbers and/or percentages of patients expressing CIMP was extracted, as were DFS and OS.

### Definition of CIMP

No consensus definition of CIMP exists across the published literature. Studies variable report a binary CIMP +ve or -ve, or groupings of CIMP-high(-H), CIMP-intermediate(-I), CIMP-low(-L), or CIMP-negative(-N). For the purposes of this review, dichotomisation of different CIMP classifications was performed so to classify results into CIMP +ve (including CIMP +ve, CIMP-high, and CIMP-intermediate groups) and CIMP -ve (including CIMP-low and CIMP -ve/-N). This process is keeping with that of other authors [21].

### Quality of studies

Two reviewers independently assessed the quality of included studies using the Newcastle-Ottawa (N-O) scale [22]. A score of 6 out of 9 criteria fulfilled on assessment of selection, comparability, and outcome was used as a cutoff for inclusion in the analysis, which was itself conducted according to PRISMA guidelines [23].

### Statistical analysis

Analysis was conducted using RevMan statistical software (v 5.3. Pub: The Cochrane Collaboration). Heterogeneity was calculated as the chi-squared value (*X*^2^, df −1) and the *I*^2^ statistic [24]. Overall effect was calculated by *Z* test with significance set as *p* < 0.05, based on meta-analysis employing Mantel-Haenszel odds ratio (OR) for CIMP status and 5-year overall survival rates with 95% confidence intervals (CI).

## Results

**Fig. 1:**
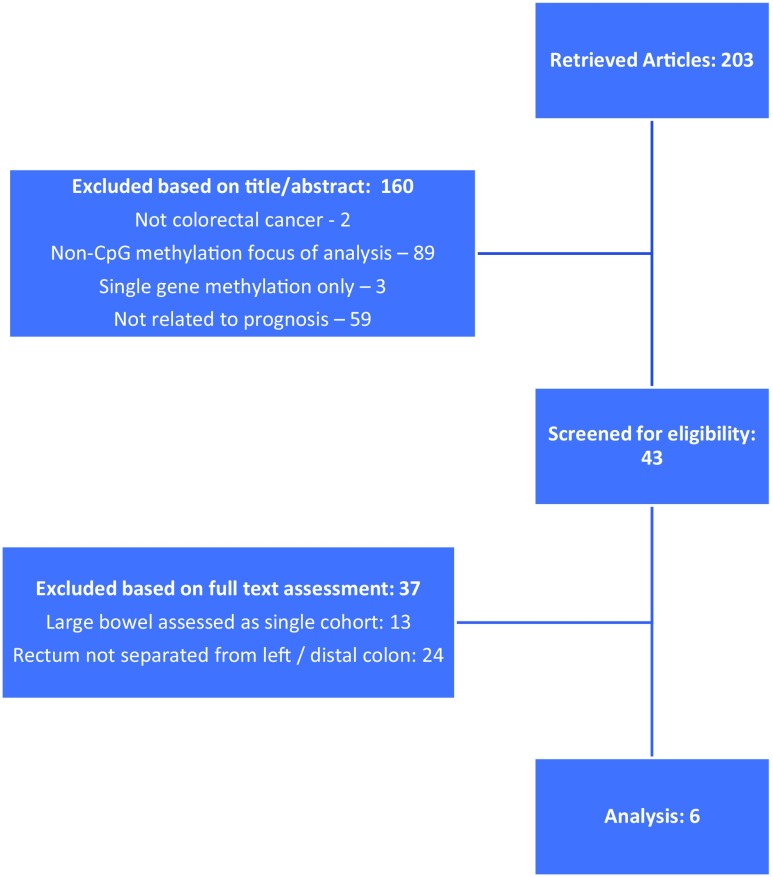
Consort diagram showing selection criteria for inclusion in the analysis

Electronic search yielded 203 original articles, once duplications had been removed. No additional studies were found in the grey literature. Following review of abstracts, 160 articles were excluded on the basis of not relating to colorectal cancer (2), CIMP not basis of analysis or not assessed (89), single locus/gene methylation only (3), survival outcomes not assessed (59), and being review articles (7). The remaining 43 articles then underwent full manuscript screening for eligibility, where a further 37 were excluded on the basis of the colon and rectum being assessed as a single cohort (13), and the rectum not being defined as an individual cohort separable from the left or distal colon (24) (Fig. 1). The remaining six studies were included, and baseline and CIMP/outcomes data extracted (Table 1) [16, 17, 25–28].

**Table 1:** Characteristics of included studies

Reference	No. of patients	Study interval	Age range (mean)	Men (%)	AJCC	nCRT	N-O score
Samowitz et al. 2009 [24]	864	1997–2001	30–79 (nr)	nr	I–IV	nr	6
Jo et al. 2011 [17]	150	2004–2006	nr (61)	71	II–IV	No	6
Bae et al. 2013 [16]	168	2004–2006	36–87 (62)	67	I–IV	No	7
Williamson et al. 2017 [25]	160	2002–2011	nr (65)	71	II–IV	Yes	7
Kim et al. 2017 [26]	87	2006–2007	31–88 (65)	59	I–IV	nr	7
Kokelaar et al. [27]	100	2010–2013	24–89 (71)	70	I–IV	No	7

*nr* not recorded/extractable

The median N-O score for the included studies was 7 (range 6–7). No studies were excluded on the basis of quality assessed by this metric. Five studies were based on retrospective cohorts and one was based on a cohort extracted from an ongoing phase III clinical trial [17]. CIMP analysis was performed on resected specimens in all but one study (Jo [17]; pre-treatment biopsies) and all employed poly-locus methodologies. Two studies assessed methylation in CRC but had extractable primary outcome data for a RC sub-cohort, although extractable clinicopathological data was often not available for this sub-cohort [16, 27]. The remaining four studies only assessed RC.

The six studies included 1529 patients with a mean sample size of 254 (range 78–864). Only one paper reported a cohort of less than 100 (Kim; 87). Each of the studies included patients with AJCC stage I–IV tumours, except Williamson and Jo (II–IV). The approximate mean age of the patients was 62–65 years, and 68% were male. Three studies specify that all patients were nCRT naïve, two make no statement regarding nCRT prior to tissue sampling, and one specifies all patients received nCRT (Table 1).

CpG island methylation status was assessed in a median of 6.5 loci (range 5–8), with a total of 15 different genes employed across all studies. CIMP characterisation is described in all papers, two employing a +ve/ve strategy and the others employing variable strategies of CIMP-H/-I/-L/-N (Table 2). Following dichotomisation, the prevalence of CIMP +ve tumours ranged from 10 to 57%, with a median of 12.5%. Two studies reported a positive association between CIMP and overall poor survival, and four reported no association.

**Table 2:** CIMP methodologies

Reference	CIMP markers	CIMP classification	CIMP association with outcome
Samowitz et al. 2009 [24]	hMLH, MINT1, MINT2, MINT31, CDKN2A	CIMP-positive vs CIMP-negative	CIMP-high poorer survival (*p* < 0.040)
Jo et al. 2011 [17]	SOCS1, RUN3, NEUROG1, IGF2, CACNA1G	CIMP-positive vs CIMP-negative	No statistical significance (*p* > 0.050)
Bae et al. 2013 [16]	hMLH1, CDKN2A, SOCS1, RUNX3, NEUROG1, IGF2, CACNA1G, CRABP1	CIMP-high vs CIMP-low vs CIMP-negative	CIMP-high poorer survival (*p* = 0.019)
Williamson et al. 2017 [25]	hMLH1, MINT1, SOCS1, NEUROG1, THBD, HAND1, ADAMTS1, IGFBP3	CIMP-high vs CIMP-intermediate vs CIMP-low	No statistical significance (*p* > 0.050)
Kim et al. 2017 [26]	SOCS1, RUN3, NEUROG1, IGF2, CACNA1G	CIMP-high vs CIMP-low vs CIMP-negative	No statistical significance (*p* > 0.050)
Kokelaar et al. [27]	hMLH1, MINT1, SOCS1, NEUROG1, THBD, HAND1, ADAMTS1, IGFBP3	CIMP-high vs CIMP-intermediate vs CIMP-low	No statistical significance (*p* > 0.050)

**Fig. 2:**
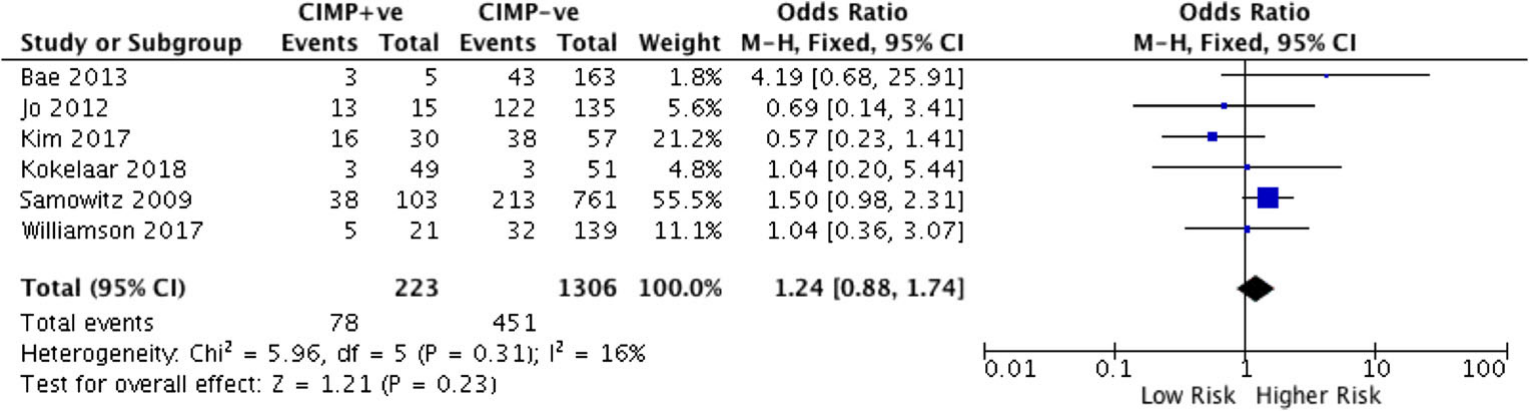
Pooled analysis between CIMP and OS

Meta-analysis demonstrated that the pooled OR for all-cause death for CIMP +ve tumours versus CIMP -ve tumours was 1.24 (95% CI 0.88–1.74). *Z* test for overall effect was 1.21 (*p* = 0.23) (Fig. 2). Heterogeneity between the studies was low (*X*^2^ 5.96, df 5, *p* = 0.31, *I*^2^ = 16%). Single and multivariate analysis of individual genes used in assessing CIMP did not demonstrate any association with outcomes. Pooled analysis of clinicopathological factors assessed in relationship to CIMP and survival was not possible due to inconsistent reporting and non-extractable data.

## Discussion

This study demonstrates the significant heterogeneity in methods used to assess CIMP in RC, but that across a range of gene panels, CIMP has not been shown to be associated with worse overall survival in rectal cancer. Confidence intervals for individual studies were broad and each range crossed OR 1.0. Of the two studies that did report an increased risk of death, the 5-year survival rates were reported as 63.2 and 25% respectively, although numbers in the second group was extremely small (*n* = 5). DFS was not reported consistently or extractably across studies and was therefore not meta-analysed, although again, there was conflicting data presented between studies. Jo reports that although there is no difference in OS, there was a statistically significant relationship between poorer DFS and CIMP-positive tumours (*p* = < 0.010), in agreement with Bae and Kim, who also report a worse DFS with CIMP-H (*p* = 0.042 and *p* = 0.018, respectively). This finding is however directly contradicted by Kokelaar, where pooled CIMP-H and CIMP-I were not associated with DFS (*p* = 0.10).

Within the broader context of CRC, CIMP has been demonstrated to be significantly related to both DFS and OS. A systematic review and meta-analysis by Juo analyses 33 studies and extracted data representing 10,635 patients, finding that the OR for DFS and OS was 1.45 (95% CI 1.07–1.97) and 1.43 (95% CI 1.18–1.73), respectively, for CIMP-positive tumours on the basis of pooled dichotomised analysis [18]. The reported prevalence of CIMP in the included studies was 4.6 to 46.5%, with a median of 18.2%. Despite the pooled analysis demonstrating a worse prognosis for DFS and OS, 8 out of 11 and 13 out of 19 studies in this meta-analysis failed to identify any significant relationship between DFS and OS, respectively. Thirty-seven studies were excluded from the analysis due to the RC sub-cohort not being presented separately from either from a whole CRC or left-sided cancer cohort, significantly limiting the power of this analysis.

The factors accounting for the differences in these observations are likely to be multifaceted, although differences in patient cohorts and lack of statistical power make interpretation difficult. In this analysis, one study relied upon a population set drawn from a randomised trial of nCRT, and thus may not be representative of the wider population [17, 29]. There was also a wide geographical variation in datasets, representing populations with likely significant differences in clinical factors such as body mass index and smoking status. Clinicopathological variables may account for some if the variation in results was not able to be sub-analysed due to data being non-extractable, frequently because it was not consistently presented across the studies or was not presented separately for rectal cancers within a larger colorectal cohort, thus making multivariable analysis impossible. These factors are keeping with the wider experience in CRC methylation research [18]. Despite none of the studies being excluded based on their respective N-O scores, the overall quality of the included studies was only good–fair, with a median score of 7 out of a possible 9.

Fifteen genes were used to assess CIMP across the 6 studies included in this analysis (median six), which is keeping with the findings of other authors. Juo reported a median of five genes in their analysis (range of three to thirteen); the most commonly employed panels being the ‘classic’ (MINT1, MINT2, MINT31, CDKN2A, and hMLH1), or the Weisenberger panel (CACNA1G, IGF2, NEUROG1, RUNX3, and SOC1) [30]. Jia and colleagues also reported that up to 15 different methylation markers were employed in studies of CRC in their systematic review of methodologies, and that the prevalence of CIMP ranged between 6.4 and 48.5% [31]. Studies investigating the relationship between a single methylated locus and survival have remain inconclusive, although hypermethylation of the promotor regions of CDNK2A and IGFBP3 has shown the greatest association with poor outcomes in some small studies, although the data is conflicting and often not replicated in studies with larger cohorts [32–34]. The relationship between CIMP and MSI is also the subject of ongoing investigation, with some evidence suggesting that methylation silencing of hMLH1 is the common factor in sporadic colorectal cancers [35]. The CIMP+/MSI+ phenotype has been associated with poor outcomes in gastric cancer based on pooled analysis of panels that included hMLH1 [21], although similar analysis in CRC has in some populations indicated the converse [36]. The complexity reflected in these studies is representative of the overlying complexity of the genetic and epigenetic profiles of CRC genetics. The Cancer Genome Atlas Network describes a pool of 125 colorectal tumours, including 62 rectal tumours that were subjected to whole-genome methylation and mutational analysis [37]. Their findings describe a complex pattern of four sub-groups characterised by overlapping but variable patterns of hypermutation and MSI [38]. However, no difference in the tumour site was noted and no outcomes were assessed relating to methylation and tumour site, contradicting other analyses [10].

## Conclusions

On the basis of this meta-analysis, there is no significant relationship between CIMP and overall survival in rectal cancers. Disease-free survival and individual clinicopathological variables were not able to be analysed due to a paucity of extractable data. No single marker for methylation drawn from within the CIMP panels included in this study significantly related to outcomes. Despite the negative findings, there is a high level of heterogeneity within CIMP panels that may account for the highly variable results. A consensus definition of CIMP and standardisation of methodologies should be agreed to progress research in this field. Additionally, it would be highly beneficial for datasets reporting outcomes in CRC to include separate cohorts specifically describing the RC sub-population so that this data is extractable for future analysis.
